# Resistance pattern of enterobacteriaceae isolates from urinary tract infections to selected quinolones in Yaoundé

**DOI:** 10.11604/pamj.2015.21.105.5469

**Published:** 2015-06-09

**Authors:** Emilia Enjema Lyonga, Michel Toukam, Celine Nkenfou, Hortense Kamga Gonsu, Marie-Claire Okomo Assoumou, Martha Tongo Mesembe, Agnes Bedie Eyoh, George Mondinde Ikomey, Valantine Ngum Ndze, Sinata Koulla-Shiro

**Affiliations:** 1 Department of Microbiology, Haematology, Parasitology and Infectious Diseases, Faculty of Medicine and Biomedical Sciences, University of Yaoundé 1, Yaoundé, Cameroon; 2 Centre for the Study and Control of Communicable Diseases, Faculty of Medicine and Biomedical Sciences, University of Yaoundé 1, Yaoundé, Cameroon; 3 Higher Teachers’ Training College, University of Yaoundé 1, Yaoundé, Cameroon; 4 Systems Biology Laboratory, Chantal Biya's International Reference Centre (CBIRC), Yaoundé, Cameroon

**Keywords:** Quinolone, enterobacteriaceae, urinary tract infections, resistance

## Abstract

**Introduction:**

It is estimated that 150 million urinary tract infections (UTIs) occur yearly worldwide, resulting in more than 6 billion dollar in direct healthcare cost. The etiology of UTIs is predictable, with *Escherichia coli*, an Enterobacteriaceae being the principal pathogen. Quinolones are usually the drug of choice. In this study, we report the resistance pattern of Enterobacteriaceae isolates from UTIs to quinolones among in-patients and out-patients at the Yaoundé Reference Hospital in Cameroon.

**Methods:**

A cross-sectional descriptive study was carried out for a ten-month period. Consecutive clean-catch mid-stream urine samples were collected from 207 in and out-patients. Identification was done using the Api 20E, and susceptibility testing using the Kirby Bauer's disc diffusion method and the MIC was done using the E-test.

**Results:**

Out of the 207 isolates, 58(28.0%) were found to be resistant to all the quinolones used in the study. The resistances observed by species were in the order: *Enterobacter* 4(30.8%); *Klebsiella* 19(29.7%); *Escherichia* 25 (29.4%); *Proteus* 2(11.8%); *Serratia* 4(25.0%). Quinolone resistance for *Escherichia* was 42.9% for In-Patients (IP) and 16.3% for Out-Patient (OP) (P-value = 0.006); *Klebsiella* 35.9% for IP and 20% for OP; *Proteus* 11.1% for IP and 12.5% for OP; *Serratia* 18.2% for IP and 40% for OP; *Enterobacter* 22.2 for IP and 50% for OP.

**Conclusion:**

High resistance rates to quinolones were observed not only for in-patients but also for out-patients with urinary tract enterobacterial infections. These findings demonstrate the importance of antibiotics susceptibility testing in improving quinolones prescription practices in Cameroon.

## Introduction

Urinary tract infections (UTIs) are common infections. It is estimated that 150 million urinary tract infections occur yearly worldwide, resulting in more than 6 billion dollar in direct healthcare cost [1]. Complicated UTIs include those in patients with stones or obstructive uropathies and in patients with catheter-related infections. These infections are often associated with nosocomial, antibiotic-resistant gram-negative and gram-positive bacteria [2]. The etiology of UTIs is predictable, with *Escherichia coli* an Enterobacteriaceae being the principal pathogen [3].

However, *E. coli* and other uropathogens are becoming increasingly resistant to commonly prescribed antimicrobials, resulting in decreased effectiveness of some standard regimes. Quinolones are the drug of choice for these infections [4]. Ciprofloxacin, ofloxacin, lomefloxacin (Maxaquin), enoxacin (Penetrex), levofloxacin, and gatifloxacin have higher renal clearance and greater renal concentrations; they are optimal choices for the treatment of complicated UTIs [2].

Quinolones were first introduced into use in 1962 in the form of nalidixic acid, which is a completely synthetic agent that in clinical concentrations has bactericidal effects on most members of the Enterobacteriaceae [5, 6]. Just as with other antimicrobials, the extensive use or administration of these compounds has led to the development of resistance by the bacteria [7–10].

Little is known about the antimicrobial resistance patterns of community-acquired organisms that circulate in developing countries where antimicrobials are available without prior consultation with a physician most often leading to auto medication [11].

Several surveillance studies from different parts of the world have shown that resistance to the fluoroquinolones among Enterobactriaceae has increased dramatically worldwide especially during the past five years [12].

In this study, we report the resistance pattern in Enterobacteriaceae isolates from urinary tract infections to quinolones among in and out-patients at the Yaoundé Reference Hospital in Cameroon

## Methods

### Specimen

A total of 207 urine specimens were collected consecutively within a period of ten months. Specimens were collected from in and out-patients at the Yaoundé General Hospital. The collection was done by trained medical personnel avoiding contamination. Clean-catch mid-stream urine samples were collected from consenting patients. The specimens were immediately transported to the laboratory after collection and processed. All contaminated urine specimens and all patients or children whose guardians refused to fill the consent form were excluded from the study.

### Isolation and identification

The specimens were inoculated onto Nutrient agar plates using a calibrated loop designed to deliver a known volume of 5µl. Inoculated plates were then incubated aerobically at 37°C for 24 hours. After 24 hours, discrete colonies were picked up and gram stained. Further sub-culturing for gram negative bacilli was done on Eosin Methylene Blue agar to obtain a pure culture and biochemical tests were carried out using the Api 20E identification kit in accordance with the manufacturer's manual ((BioMérieux SA, Lyon, France).

### Antimicrobial susceptibility testing

This was done using the Kirby Bauer Disc diffusion method with reference to the Clinical Laboratory Standard Institute (CLSI) performance guideline for antimicrobial susceptibility testing [13]. Quality was assured by testing the E. coli quality control strain, ATCC 25922, in every batch. All zones of inhibition determined were within the ranges prescribed by the CLSI. Seven quinolones were used; nalidixic acid (NA) and pipemidic acid (PI) of the first generation; ciprofloxacin (CIP), norfloxacin (NOR), and ofloxacin (OFX) of the second generation; sparfloxacin (SPX) of the third generation; and moxifloxacin (MXF) of the fourth generation.

### Minimum inhibitory concentration (MIC)

The minimum inhibitory concentration was determined for all the isolates that were found to be resistant in the disc diffusion method using the e-test. Ciprofloxacin was used. The method was controlled by parallel testing using quality control reference strain (ATCC 25922)) as recommended by the CLSI

### Ethical considerations

This study received approval from the ethics committee of the Faculty of Medicine and Biomedical Sciences of the University of Yaoundé 1. All the patients signed the consent form before specimen collection. All the samples were assigned codes after collection.

## Results

From the 207 urine specimens collected, 118 (57%) isolates were from in-patients and 89 (43%) from out patients. The prevalence of Enterobacteriaceae in the isolates was in the order: Escherichia 85 (41.1%); *Klebsiella* 64(30.9%); *Proteus* 17(8.2%); *Serratia* 16(7.7%); *Enterobacter* 13(6.3%); others (*Citrobacter, Kluyvera, and Morganella*) 12(5.9%)

**Figure 1:**
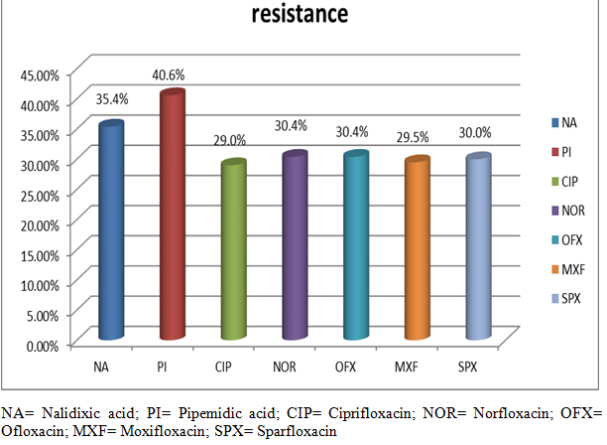
The resistance rate of the enterobacteriaceae isolates to the selected quinolones

Out of the 207 isolates, 58 (28.0%) were found to be resistant to all the quinolones. The resistance observed in each species were in the order:*Enterobacter* 4(30.8%); *Klebsiella* 19(29.7%); *Escherichia* 25 (29.4%); *Proteus*2(11.8%); *Serratia* 4(25.0%); others 4(33.3%) Figure 1. The resistance of each quinolone was NA 74(35.4%); PI 84 (40.6%); CIP 60(29.0%); OFX 63(30.4); NOR 63 (30.4%); SPX 62(30.0%) and MXF 61(29.5%) (Shown inFigure 1) Table 1.

**Table 1:** The prevalence of the enterobacteriaceae isolates in hospitalized and community patients

Species	In-patients (N = 118)		Out-patients (N = 89)		p-value
	No	%	No	%	
*Escherichia*	42	35.6%	43	48.3%	0.066
*Klebsiella*	39	33.1%	25	28.1%	0.445
*Proteus*	9	7.6%	8	9.0%	0.764
*Serratia*	11	9.3%	5	5.6%	0.323
*Enterobacter*	9	7.6%	4	4.5%	0.358
others	8	6.8%	4	4.5%	0.3
**Total**	**118**	**57.0%**	**89**	**43%**	**0.765**

The prevalence of all the Enterobacteriaceae in the in-patients (IP) was 118/207(57.0%) and in out-patients (OP) was 89/207(43%) (See Table 1). The prevalence of *Escherichia* was 42/118(35.6%) for IP and 43/89(48.3%) for OP, for *Klebsiella* 39/118(33.1%) for IP and 28/89(28%) for OP, for Proteus 9/118(7.6%) for IP and 8/89(9%) for OP, for *Serratia* 11/118(9.3%) for IP and 5/89(5.6%) for OP, for *Enterobacter* 9/118(7.6%) for IP and 4/89(4.5%) for OPTable 2.

**Table 2:** The resistance of the enterobacteriaceae to quinolones

Species	In-patients (n = 118)		Out-patients (n = 89)		p-value
	No Resistant	%	No resistant	%	
*Escherichia*	18	42.9%	7	16.3%	0.006
*Klebsiella*	14	35.9%	5	17.9%	0.13
*Proteus*	1	11.1%	1	12.5%	0.335
*Serratia*	2	18.2%	2	40%	1.0
*Enterobacter*	2	22.2%	2	50%	1.0
others	4	50%	1	25%	>0.3
Total	41	34.75%	18	20.23%	

The resistance of all the Enterobacteriaceae in the in-patients (IP) was 41/118(34.75%) and in out-patients (OP) was 18/89(20.23%) as shown onTable 2. The resistance in *Escherichia* was 18/42(42.9%) for IP and 7/43(16.3%) for OP, for *Klebsiella* 14/39(35.9%) for IP and 5/28(17.9%) for OP, for *Proteus* 1/9(11.1%) for IP and 1/8(12.5%) for OP, for *Serratia*2/11(18.2%) for IP and 2/5(40%) for OP, for *Enterobacter* 2/9(22.2%) for IP and 1/4(25%) for OP.

## Discussion

In this study we describe the resistance pattern of Enterobacteriaceae isolates from urinary tract infections to a selected number of quinolones among in and out-patients. The overall resistance of the Enterobactericeae to these quinolones was high. In African countries with high infectious disease burden, formal and informal health systems depend heavily on broad spectrum orally-administrable antibacterial. This has caused an increase in the resistance of these antibacterial over the years and is also affecting even the newer antibacterial like the fluoroquinolones [14].

Quinolones are widely used for the treatment of serious *E. coli* Urinary tract infections (UTIs) and may also be used to treat other infections caused by other members of the Enterobacteriaceae family. Hence, these high levels of quinolone resistance may lead to treatment failure which is of significant concern. These high rates of fluoroquinolone resistance observed could also limit treatment options especially oral treatment [15, 16]. The statistically significance difference in resistance between the in and out-patients could be due to hospitalized patient being infected by nosocomial bacteria.

The strength of this study is the broad population of interest that is both the in and out-patients reflecting the circulating resistance. There are however some limitations. Our study had a relatively small sample size. We also carried out susceptibility testing on only a limited numbers of quinolones using only two first generations, three second generation, one third generation and one fourth generation. This might not be representative of the whole class of quinolones which includes several quinolones in each generation.

The overall resistance of 28% to all selected quinolone was slightly higher than what was found in other studies carried out in Ghana, Greece, the Netherlands and Brazil [16–19]. The resistant rate of the isolate from inpatients was higher than that from the outpatients. This is similar to what was found in other studies [17]. In our study we found that the *Escherichia* species were the most resistant among the inpatient whereas other studies carried out in Greece [17] found *Klebsiella* to be the most resistant. However, among the out-patient *Klebsiella* was classified as the most resistant just as found in other studies. There was a statistically significant difference between the overall resistance in inpatients and out-patients just as also shown by another study [17].

Many factors have contributed to this high resistance rates; excessive antibiotic prescription is related to a higher prevalence of antibiotic resistant bacteria, misuse of antibiotics by health professional, unskilled practioners and laypersons (antibiotics can be purchased without a prescription). Poor drug quality, unhygienic conditions and inadequate surveillance also account for the spread of resistant bacteria [20–23].

Another area to be exploited for future research is the genetic diversity and the characterization of these quinolone resistant Enterobacteriaceae strains. This will enable us determine the prevalence of plasmid-mediated resistance which is a common phenomenon among quinolone resistant strains. Further work may also identify the prevalence of expanded spectrum beta-lactamases among these quinolone resistant Enterobacteriaceae strains.

## Conclusion

High resistance rates to quinolones were observed not only for in-patients but also for out-patients with urinary tract enterobacterial infections. These findings demonstrate the importance of antibiotics susceptibility testing in improving quinolones prescription practices in Cameroon.

## References

[1] Stamm WE, Norrby RS (2001). Urinary Tract infections: Disease panorama and challenges. Journal of Infectious Diseases.

[2] Oliphant Catherine M, Green Gary M (2002). Quinolones: a Comprehensive Review. American Family Physician.

[3] Karlowsky JA, Jones ME, Thornsberry C, Critchley I, Kelly LJ, Sahm DF (2001). Prevalence of antimicrobial resistance among urinary tract pathogens isolated from female outpatients across the US in 1999. International Journal of Antimicrobial Agents..

[4] Arslan Hande, Azap Ozlem Kurt, Ergönül Onder, Timurkarynak Funda (2005). Risk factors for ciprofloxacin resistance among Escherichia coli strains isolated from urinary tract infecttions in Turkey. Journal of Antimicrobial Chemotherapy.

[5] Andriole Vincent T An overview of fluoroquinolones focus on moxifloxacin. Modern Medicine.

[6] Xiang Chen, Weijuan Pan, Weijiu Zhang, Zhiming Pan, Song Gao, Xinan Jiao (2011). Quinolone resistance in Escherichia coli and Salmonella spp: Isolates from diseased chickens during 1993-2008. African journal of Microbiology Research.

[7] Ball Peter (2000). Quinolone Generations: Natural History or Natural Selection. Journal of Antimicrobial Chemotherapy.

[8] Bearden David T, Danziger Larry H (2001). Mechanisms of action of Resistance to Quinolones. Pharmacotherapy.

[9] King Dana (2000). New classification and update of the quninolone antibiotics. American Family Physician.

[10] Paauw Armand, Fluit Ad C, Veroef Jan, Leverstein-van Hall Maurine A (2006). Enterobacter cloacae outbreak and emergence of quinolone resistance gene in Dutch hospital. Emerging Infectious Diseases.

[11] Minh Vien Le Thi, Baker Stephen, Phuong Thao Le Thi, Phuong Tu Le Thi, Thu Thuy Cao, Thu Nga, Minh Hoang Nguyen Van, Campbell Lain James, Minh Yen Lam, Trong Hieu Nguyen, Vinh Chau NguyenVan, Farrar Jeremy, Schultsz Constance (2009). High prevalence of plasmid-mediated quinolone resistance determinants in commensal members of the enterobacteriaceae in Ho Chi Minh City, Vietnam. Journal of Medical Microbiology.

[12] Pitout Johann DD, Wei Yi, Church Deirdre L, Gregson Daniel B (2008). Surveillance for Plasmid Mediated Resistance in Enterobacteriaceae within the Calgary Health Region, Canada: the emergence of aac(61)Ib-cr. Journal of Antimicrobial Chemotherapy..

[13] Clinical Laboratory Standard Institute (2007). Performance Standards for Antimicrobial Susceptibility Testing.

[14] Namboodiri Seerla, Opintan Japheth A, Lijek Rebeccah S, Newman Mercy J, Okeke Iruka N (2011). Quinolone resistance in Escherichia coli from Accra, Ghana. BMC Microbiology.

[15] Paterson David L (2006). Resistance in Gram-negative bacteria: Enterobacteriaceae. Am J Infect Control.

[16] van der Starre Willize E, van Nieuwkoop Cees, Paltansing Sunita, van't Wout Jan W, Groenveld Geert H, Becker Martin J, Koster Ted, Wattle-Louis Hanke G, Delfos Nathalie M, Ablij Hans C, Leyten Eliane MS, Blom Jeanet W, van Dissel Jaap T (2011). Risk factors for fluoroquinolone-resistant Escherichia coli in adults with community-onset febrile urinary tract infection. J Antimicrob Chemother.

[17] Skandemi-Epitropaki V, Xanthaki A, Tsiringa A. Fotiou P, Kontou CHA, Toutouza M (2006). Fluoroquinolone resistance in Enterobacteriaceae strains isolated from community-acquired urinary tract infections. European Society of Clinical Microbiology and Infectious Disease.

[18] Ito Carmen Antonia Sanches, Gales Ana Christina, Tognim Maria Christina B, Munerato Patricia, Costa Libera Maria Dalla (2008). Quinolone-Resistant Escherichia coli. Brazilian Journal of Infectious Diseases.

[19] Hortat F, Muntean D, Hogea E, Horthat D, Craciunescu M, Licker M, Rosca A, Baditoiu L, Moldovan R (2010). Quinolone resistant enterobacteriaceae strains isolated from urinary tract infections in the intensive care unit. Journal of experimental Medical and surgical Research..

[20] Pieboji Joseph Gangoue, Koulla-shiro Sinata, Ngassam Pierre, Adiogo Dieudonne, Njine Thomas, Ndumbe Peter (2004). Antimicrobial resistance of Gram-negative bacilli isolates from inpatients and outpatients at the yaounde central Hospital, Cameroon. International Journal of Infectious Diseases.

[21] Toukam M, Lyonga EE, Assoumou M-CO, Fokunang CN, Atashili J, Kechia AF, Gonsu HK, Mesembe M, Eyoh A, Ikomey G, Akongnwi E, Ndumbe P (2010). Quinolone and fluoroquinolone resistance in Enterobacteriacea isolated from hospitalized and community patients in Cameroon. Journal of Medicine and Medical Sciences.

[22] Lyonga EE, Toukam M, Atashili J, Gonsu HK, Adiogo D, Mesembe M, Nguefack- Tsague G, Eyoh A, Ikomey G, Mukwele B, Meli Tiabou JM, Okomo Assoumou MC (2013). A comparative study of the susceptibility of six quinolones in Yaounde. Health Science and Disease..

[23] Sundvall Par-Daniel, Elm Marie, Gunnarsson Ronny, Molstad Sgvard, Rodhe Nils, Jonsson Lars, Ulleryd Peter (2014). Antimicrobial resistance in urinary pathogens among Swedish nursing home residents remains low: a cross-sectional study comparing antimicrobial resistance from 2003 to 2012. BMC Geriatrics.

